# New somatic *BRAF* splicing mutation in Langerhans cell histiocytosis

**DOI:** 10.1186/s12943-017-0690-z

**Published:** 2017-07-06

**Authors:** Sébastien Héritier, Zofia Hélias-Rodzewicz, Rikhia Chakraborty, Amel G. Sengal, Christine Bellanné-Chantelot, Caroline Thomas, Anne Moreau, Sylvie Fraitag, Carl E. Allen, Jean Donadieu, Jean-François Emile

**Affiliations:** 1 French Reference Center for Langerhans Cell Histiocytosis, Trousseau Hospital, Assistance Publique–Hôpitaux de Paris, Paris, France; 2 EA4340, Versailles SQY University, Paris-Saclay University, Boulogne, France; 3 Department of Pediatric Hematology and Oncology, Trousseau Hospital, Assistance Publique–Hôpitaux de Paris, Paris, France; 4 Pathology Department, Ambroise Paré Hospital, Assistance Publique–Hôpitaux de Paris, Boulogne, France; 5 Texas Children’s Cancer Center, Texas Children’s Hospital, Houston, TX USA; 6 Division of Pediatric Hematology-Oncology, Department of Pediatrics, Baylor College of Medicine, Houston, TX USA; 7 Department of Genetics, Pitié-Salpétrière Hospital, Assistance Publique–Hôpitaux de Paris, Paris, France; 8 Department of Pediatric Hematology and Oncology, Centre Hospitalo-Universitaire de Nantes, Nantes, France; 9 Pathology Department, Centre Hospitalo-Universitaire de Nantes, Nantes, France; 10 Pathology Department, Necker Hospital, Assistance Publique–Hôpitaux de Paris, Paris, France

**Keywords:** Langerhans cell histiocytosis, BRAF, Splicing mutation, Targeted therapy

## Abstract

Langerhans cell histiocytosis (LCH) is an inflammatory myeloid neoplasia with constitutive activation of the MAPKinase RAS-RAF-MEK-ERK cell signaling pathway. We analyzed 9 LCH cases without *BRAF* ^V600^ and *MAP2K1* mutations by whole exome sequencing. We identified a new somatic *BRAF* splicing mutation in 2 cases. Both cases were childhood single system (SS) LCH cases, with self-healing outcome of the bone lesions. This mutant consisted in a 9 base pair duplication (c.1511_1517 + 2 duplication), encoding for a predicted mutant protein with insertion of 3 amino acids (p.Arg506_Lys507insLeuLeuArg) in the N-terminal lobe of the kinase domain of BRAF. Transient expression of the c.1511_1517 + 2dup *BRAF* mutant in HEK293 cells enhanced MAPKinase pathway activation, and was not inhibited by vemurafenib but was inhibited by PLX8394, a second-generation BRAF inhibitor able to inhibit signaling of BRAF monomers and dimers. Future LCH molecular screening panel should include this new mutation to better define its prevalence in LCH and its restriction to autoregressive bone SS LCH.

**Electronic supplementary material:**

The online version of this article (doi:10.1186/s12943-017-0690-z) contains supplementary material, which is available to authorized users.

## Background

Langerhans cell histiocytosis (LCH) is the most common histiocytosis, and is characterized by inflammatory lesions containing abundant CD1a + CD207+ histiocytes that lead to the destruction of affected tissues [1]. A *BRAF* ^V600E^ mutation, responsible for activation of the MAPKinase RAS-RAF-MEK-ERK cell signaling pathway in pathologic histiocytes, is present in ~55% of LCH cases and was associated with recurrence and high-risk presentation [2]. Responses to BRAF inhibitors in patients with *BRAF* ^V600E^-mutated LCH confirms that *BRAF* ^V600E^ is a driver mutation in LCH [3]. Although ~45% do not have *BRAF* ^V600E^ mutation, ERK was reported to be activated in pathologic histiocytes of all LCH samples [4]. Other molecular alterations have also been reported to activate the MAPKinase pathway in *BRAF* ^V600E^-non mutated LCH, such as *MAP2K1* mutations (10-20% of LCH) [5, 6], β3-αC loop deletion in the kinase domain of BRAF (6% of LCH) [7], and case reports highlighted mutation on *ARAF* [8] and *MAP3K1* [9]. Fusion events involving *BRAF* and activating MAPkinase pathway have also been reported in histiocytoses of the L group [7, 10].

To identify the mechanism of pathologic ERK activation in the remaining LCH, we performed whole exome sequencing (WES) on selected LCH frozen biopsy samples wild-type for the most common activating mutations reported in LCH. DNA extracted from peripheral white blood cells (PBMC) were used as the “normal” sample for comparison. Mutation function and response to MAPKinase pathway inhibitors were assessed using in vitro constructs.

## Results

From the French LCH registry [11], 9 patients fulfilled the following inclusion criteria: i) fresh frozen biopsy tissue and blood samples available, ii) high percentage of lesions-infiltrating CD207+ histiocytes (>30%), iii) no mutation identified by *BRAF* ^V600E^ pyrosequencing [2] or among the most common activating mutations of *PIK3CA*, *BRAF*, *KRAS* and *NRAS* with the i-plex mass spectrometric based genotyping technology (Sequenom-Agena Bioscience) [12], iv) negative screening for exon 2-3 *MAP2K1* mutations by Sanger sequencing. Among the 9 included patients, 7 had a bone-limited LCH and 2 had a LCH involving several organs **(**Table 1).

**Table 1:** Clinical data and sequencing results for LCH cases without *BRAF* ^V600E^ or *MAP2K1* mutations

Patient	Gender	Age at diagnosis (years)	Extension (DAS max)	Involved organs	First-line therapy	Response to first-line therapy	Recur-rence	MAPKinases mutation^a^
P1	F	1.0	MS RO+ (5)	Multifocal bone, lymph nodes, hematologic, liver, spleen	VLB-steroid	ADB	No	WT
P2	M	17.5	SS (1)	Localized bone	Wait and see	_	No	WT
P3	F	0.8	MS RO+ (8)	Multifocal bone, skin, lymph nodes, hematologic, spleen	VLB-steroid	ADB	Yes	WT
P4	M	0.5	SS (1)	Localized bone	VLB-steroid	NAD	No	WT
P5	F	4.9	SS (1)	Localized bone	Wait and see	_	No	BRAF c.1511_1517 + 2dup
P6	F	10.2	SS (0)	Multifocal bone	Wait and see	_	Yes	BRAF c.1511_1517 + 2dup
P7	F	8.7	SS (0)	Localized bone	Wait and see	_	No	WT
P8	F	1.1	SS (1)	Multifocal bone	VLB-steroid	NAD	No	WT
P9	M	2.8	SS (4)	Localized bone	VLB-steroid	NAD	No	WT

*ADB* active disease better, *DAS max* maximum Disease Activity Score (DAS) measured during the clinical course for each patient, *F* female, *M* male, *MS RO+* multiple systems LCH with risk organs involvement, *NAD* non-active disease, *SS* single system LCH, *VLB* vinblastine

^a^Deleterious coding missense or non-sense or small indel mutations in genes involved in the MAPkinase cell signaling pathway

### Detection of duplication at the end of BRAF exon 12 in LCH samples

**Fig. 1:**
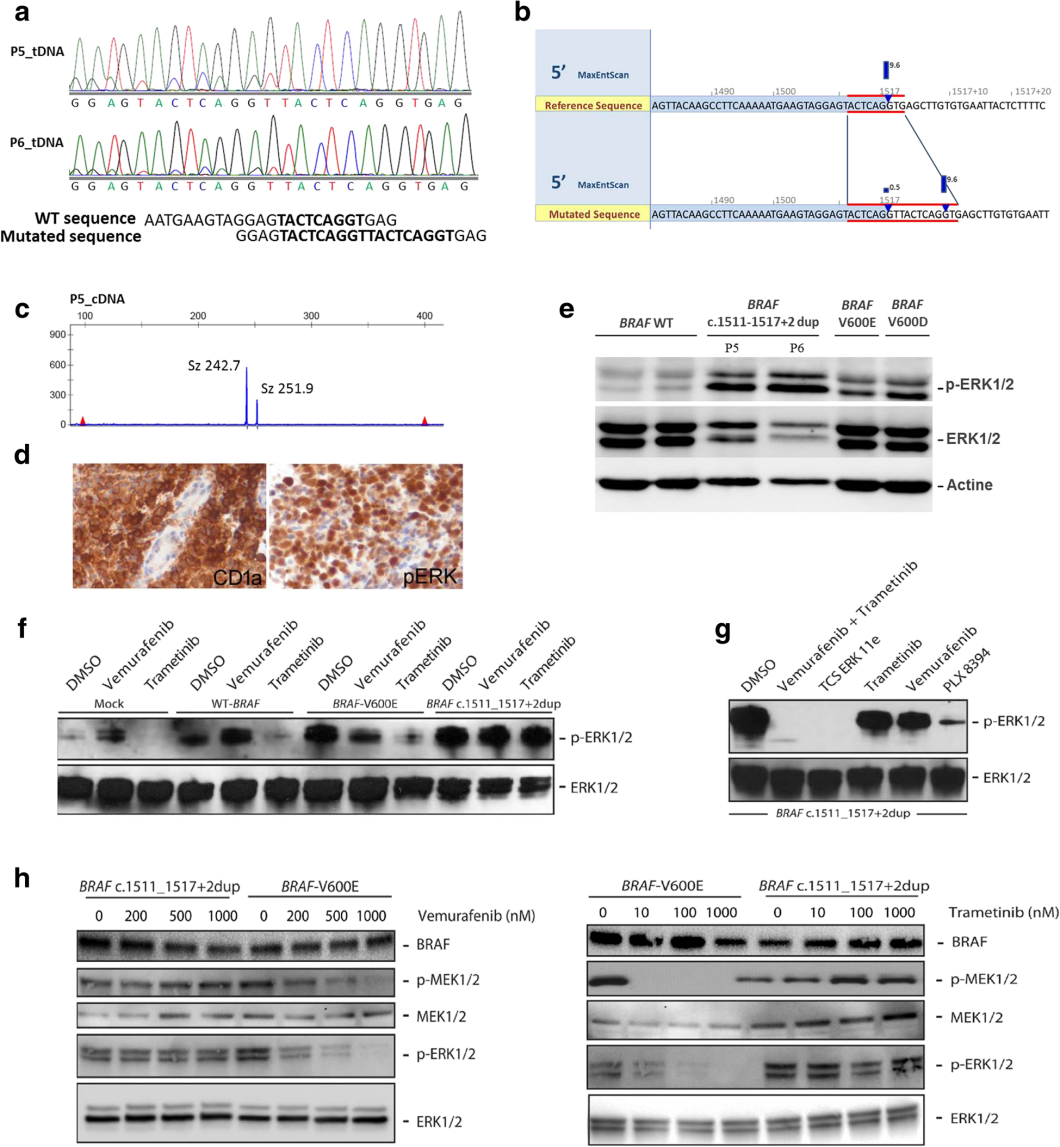
Analysis of LCH samples. **a** Sanger sequencing of P5 and P6 LCH samples shows duplication of the c.1511_1517 + 2 sequence. **b** In silico analysis (Alamut® Visual, hg19) predicts a 5*′* splice site change, causing the insertion of 9 nucleotides in the cDNA sequence [GTTACTCAG] at the end of exon 12. **(C)** P5 cDNA analyse confirms insertion of 9 nucleotides by rt.-PCR product length analysis. **d** Immunohistochemistry performed on FFPE samples from P5 showed a strong cytoplasmic and nuclear positivity of histiocytes with phosphoERK1/2 (D13.14.4E, Rabbit mAb, Cell Signaling) in areas containing numerous CD1a + LCH cells. **e** Results of the western blot (p- and total-ERK1/2) for P5 and P6 LCH. Protein extracts from two *BRAF* wild type, a *BRAF* ^V600E^-mutated LCH and a *BRAF* ^V600D^-mutated LCH were used as positive control for p-ERK. Functional analysis of the *BRAF* c.1511_1517 + 2 duplication. HEK293 cells were transiently transfected with expression plasmids encoding *BRAF* wild-type, *BRAF* ^V600E^ and *BRAF* c.1511_1517 + 2dup mutant cDNAs, and corresponding lysates from cells maintained in serum were subjected to immunoblotting with the indicated antibodies. **f** Where indicated, cells were treated with inhibitor of BRAF^V600E^ (vemurafenib) or MEK (trametinib) for 4 h before harvest. **g** Where indicated, cells were treated with combination of vemurafenib and trametinib, or with inhibitors of BRAF (PLX8394) or ERK (TCS ERK 11e) for 4 h before harvest. **h** To test dose response to vemurafenib and trametinib on *BRAF* ^V600E^ and *BRAF* c.1511_1517 + 2dup transfected cells, the cells were treated for 4 h with the specified agents (vemurafenib or trametinib) at the specified doses before harvest

A somatic duplication of 9 base pairs at the end of exon 12 of *BRAF* (nucleotides c.1511_1517 + 2) was detected in LCH samples from 2 patients (P5 and P6). Both patients were children with self-healing bone lesions. This duplication was not yet reported in the COSMIC database. For both patients, Sanger sequencing of genomic DNA confirmed the *BRAF* c.1511_1517 + 2 duplication in LCH lesions (Fig. 1a), but failed to detect it within PBMC. This 9 nucleotides insertion at the position +2 of the splice donor site of intron 12 was predicted to change the splicing, with an insertion of 9 nucleotides in the cDNA sequence [GTTACTCAG] at the end of exon 12 (Fig. 1b). Messenger RNA was extracted from lesion of P5, and length analysis of PCR products of cDNA confirmed a 9 nucleotides insertion (Fig. 1c). Insertion was also confirmed by Sanger sequencing (Additional file 1: Figure. S1).

To investigate the prevalence of the somatic *BRAF* c.1511_1517 + 2 duplication in LCH, we studied 28 additional LCH samples wild-type for *BRAF* ^V600^, by length analysis of PCR products. No additional mutated case was found, suggesting that this mutation represents a small proportion of *BRAF* ^V600^ wild-type LCH (<10%) [7], but more studies are needed to estimate precisely its prevalence.

### Functional analysis and response to MAPkinase inhibitors

The insertion of 3 amino acid (p.Arg506_Lys507insLeuLeuArg) coded by this 9 base pair duplication is localized in the smaller N-terminal lobe of the kinase BRAF domain responsible for ATP binding (Additional file 1: Figure. S2). Small deletions localized nearby this region of *BRAF* were shown to induce MAPkinase pathway activation in LCH and in pancreatic carcinomas [7, 13], suggesting that this new mutation may also have functional impact. Immunohistochemistry of samples of P5 and P6 confirmed that areas rich in CD1a + histiocytes contained numerous histiocytes with phosphoERK in their cytoplasms as well as translocated into the nucleus (Fig. 1d). The strong phosphorylation of ERK in LCH lesions of P5 and P6 was also confirmed by Western blot (Fig. 1e).

We then assessed the functional impact of this genomic alteration on *BRAF* signaling by analyzing phosphorylation of ERK in HEK293 cells transiently transfected with wild-type *BRAF*, *BRAF* ^*V600E*^ or *BRAF* c.1511_1517 + 2dup mutants. cDNA expression of *BRAF* c.1511_1517 + 2dup, but not wild-type *BRAF*, resulted in a significant increase in ERK1/2 phosphorylation (Fig. 1f).

We also evaluated the ability of the BRAF^V600E^ inhibitor vemurafenib and the MEK inhibitor trametinib to suppress ERK activation by specific *BRAF* alterations. Although vemurafenib induced a substantial inhibition of *BRAF* ^*V600E*^-induced activation, this drug did not inhibit the MAPkinase activation in cells transfected with the cDNA containing the *BRAF* c.1511_1517 + 2dup mutant, which is consistent with specific activity of this agent against mutations that result in active BRAF monomers. Trametinib, which blocks active MEK, decreased activation of ERK in cells transfected with *BRAF* ^V600E^, but with no impact on cells transfected with the *BRAF* c.1511_1517 + 2dup mutant (Fig. 1f). We thus further evaluated the effects of other, or combination of inhibitors of the MAPkinase pathway. As expected TCS ERK 11e, which directly inhibits ERK, induced a total extinction of ERK phosphorylation (Fig. 1g). PLX8394 is a second-generation BRAF inhibitor able to inhibit signaling of BRAF monomers and dimers without paradoxical activation of MAPKpathway signaling in cells with wild-type BRAF that has been observed in first-generation agents such as vemurafenib [14, 15]. PLX8394 induced an almost complete extinction of pERK signal on Western blot, confirming that most of the pathway activation was due to the mutant *BRAF*. Again vemurafenib or trametinib alone did not suppress ERK activation, but combination of both drugs induced a completed extinction of the pERK signal (Fig. 1g).

To elucidate this last observation, we performed a dose response experiment with vemurafenib and trametinib on *BRAF* c.1511_1517 + 2dup transfected cells and *BRAF* ^V600E^ transfected cells, in order to test if an increased dose for trametinib was required to block activation by the *BRAF* c.1511_1517 + 2dup mutation as compared to other LCH-associated *BRAF* mutations. In our model, while vemurafenib and trametinib induced an inhibition of *BRAF* ^*V600E*^-induced activation with relationship between dose and response, these drugs did not inhibit the MAPkinase activation in *BRAF* c.1511_1517 + 2dup transfected cells regardless of dose (Fig. 1h). Neither vemurafenib nor trametinib used individually, even at the highest concentration, could inhibit phosphorylated MEK1/2 and phosphorylated ERK1/2. Future study should better define mechanisms of resistance of the *BRAF* c.1511_1517 + 2dup mutation by targeted therapies such as vemurafenib and trametinib.

## Conclusions

We report here a new somatic *BRAF* splicing mutation in LCH, leading to the insertion of 3 amino acids (p.Arg506_Lys507insLeuLeuArg) in the N-terminal lobe of the kinase domain of BRAF. This mutation constitutively activates the MAPKinase pathway, and was inhibited by the second-generation BRAF inhibitor PLX8394. Thanks to recent substantial effort of LCH expert teams, the unknown part of the molecular spectrum of LCH continues to shrink and identification of these mutations has many potential applications such as targeted therapy, therapeutic risk-stratification based on tumor genotype, and quantitative detection of mutant allele in circulating cell free DNA as possible blood biomarkers.

## Additional file

Additional file 1: Supplemental methods and data. (DOCX 904 kb)

## Abbreviations

ADB: Active disease better
DAS: Disease activity score
F: Female
LCH: Langerhans cell histiocytosis
M: Male
MS RO+ LCH: Multiple systems LCH with risk organ involvement
NAD: Non-active disease
PBMC: Peripheral white blood cells
SS LCH: Single system LCH;
VLB: Vinblastine
WES: Whole exome sequencing
