# Supplementary material for: New somatic BRAF splicing mutation in Langerhans cell histiocytosis
Source: Mol Cancer. 2017 Jul 6;16:115. doi: 10.1186/s12943-017-0690-z (PMC5498996; doi:10.1186/s12943-017-0690-z)
Supplement: Additional file 1: — Supplemental methods and data. (DOCX 904 kb) [file 12943_2017_690_MOESM1_ESM.docx]

**SUPPLEMENTAL METHODS AND DATA**

**METHODS**

Sanger sequencing of DNA was performed as previously described [1]. Splicing variant analysis was performed by cDNA sequencing obtained after RT-PCR from tumor-derived RNA.

Western blot and immunohistochemistry on LCH samples were performed as previously described [2, 3].

**Whole Exome Sequencing**

For the Whole Exome Sequencing, library preparation, capture, sequencing, variant detection and annotation, were performed by IntegraGen (Evry, France).

DNA extracted from peripheral white blood cells (PBMC) were used as the germinal sample for comparison with DNA extracted from LCH samples. For the 2 cases with multisystemic LCH, the blood sampling was performed more than 2 years after complete remission to avoid the possible contamination by circulating mutated cells as reported in active MS LCH [4].

5 µg of each genomic tumor and germinal DNA sample was fragmented by sonication and purified to yield fragments of 150–200 base pair in length. Paired end adaptor oligonucleotides from Illumina were ligated on repaired A-tailed fragments that were purified and enriched by six polymerase chain reaction (PCR) cycles. Purified libraries (500 ng) were hybridized to the Sure Select oligonucleotide probe capture library (SureSelect Clinical Research Exome, Agilent) for 24 h. After hybridization, washing and elution, the eluted fraction was PCR-amplified (10 to 12 PCR cycles), purified and quantified by quantitative PCR to obtain sufficient amounts of DNA template for downstream applications. Each eluted enriched DNA sample was then sequenced on an Illumina HiSeq 5000 as paired-end 75-base reads. Image analysis and base calling were performed using the Illumina Real-Time Analysis Pipeline version 1.14 with default parameters. With this sequencing protocol, samples achieved an average coverage of 111X, with 90.3% of the targeted exome bases covered to a depth of 25X or greater.

Bioinformatics analysis of sequencing data was based on the Illumina pipeline (CASAVA 1.8). CASAVA aligns reads to the human reference genome (hg19) with the alignment algorithm ELANDv2 (it performs multispeed and gapped alignments), calls SNPs on the basis of allele calls and read depth, and detects variants (SNPs and indels). Only positions included in the bait coordinates were conserved. Genetic variation was annotated with the IntegraGen in-house pipeline, consisting of gene annotation (using RefSeq), detection of known polymorphisms (using ExAC, dbSNP132, the 1000 Genomes Project, Exome Variant Server and HapMap database) and characterization of mutations as intronic or exonic, and silent, nonsense, missense and frame-shifting. Somatic variants were identifying from matched tumor/normal sample pair from each patient. Mutations interpretation was assessed with Alamut® Visual 2.7 (Interactive Biosoftware, France). The effects of amino acid substitutions on protein function were predicted using Mutation Taster, SIFT and Polyphen2. The effects of splice variants were analyzed using MaxEnt score.

***BRAF* c.1511_1517+2 dup study by PCR product length analysis**

We investigated the prevalence of the *BRAF* c.1511_1517+2 dup on *BRAF*^V600E^ non-mutated LCH by a length analysis of PCR products. PCR products were obtained from LCH derived DNA after a designed PCR encompassing the *BRAF* c.1511_1517 sequence (Table S1). PCR product length were analyzed using *ABI PRISM 3100 sequencer* (Applied Biosystems, Foster City, CA)

As for P5 and P6, LCH biopsies were conserved as fresh frozen condition and also Formalin-fixed, paraffin-embedded (FFPE) condition, we assessed the detection of the *BRAF* c.1511_1517+2 dup on DNA derived from both storage conditions. Also, LCH DNA from P1-P4 and P7-P9 which were negative for the *BRAF* c.1511_1517+2 dup by WES were also tested. Only P5 and P6 LCH samples showed positive detection with the appearance of a new peak that corresponds to a 9pb longer PCR product, observed for fresh frozen and FFPE derived DNA (**Figure S3**). A set of 28 additional *BRAF*^V600^-non mutated LCH from the French children cohort (P10-P37, **Table S2**) were also investigated. All DNA were obtained from FFPE biopsy previously investigated for the *BRAF*^V600^ mutation. PCR product length analysis failed to highlight additional *BRAF* c.1511_1517+2 dup mutated cases.

**Functional Analysis of *BRAF* Mutations** [5, 6]

***Expression constructs***

Specific mutations were generated by QuickChange XL Site-Directed Mutagenesis Kit (Agilent Technologies, La Jolla, CA) in the full length *BRAF* (#40775) vector (Addgene, Cambridge, MA) as previously described. Expression plasmids encoding mutant (V600E) *BRAF* (#17544) were obtained from Addgene.

***Cell culture, transfection and treatment***

HEK293 cells (1.5 x 10^6^) (ATCC, Manassas, VA), cultured in DMEM (Lonza, Walkersville, MD) supplemented with 10% FBS (Lonza), and 2 mM L-glutamine (Life Technologies, Carlsbad, CA), were transfected using Lipofectamine 3000 (Life Technologies, Carlsbad, CA). Where indicated, the cells were treated for 4 hours with either 200 nM of the BRAF inhibitor vemurafenib (Roche, Basel, Switzerland), or 10 nM of the MEK inhibitor trametinib (Selleckchem, Houston, TX), or 40 nM of the ERK inhibitor TCS ERK 11e (Tocris Bioscience, Bristol, UK) or 1μM of the pan-BRAF inhibitor PLX-8394 (Plexxikon, Berkeley, CA), before harvesting. Cells were harvested 48 hours post-transfection.

To test dose response to vemurafenib and trametinib on *BRAF*^V600E^ and BRAF c.1511_1517+2dup transfected cells, the cells were treated for 4 hours with vemurafenib or trametinib at the specified doses (0-1000 nM) before harvest.

***Immunoblot analysis of MEK and ERK***

Harvested HEK293 cells were lysed in M-PER Mammalian Protein Extraction Reagent (Thermo Scientific, Waltham, MA) containing Halt protease inhibitor cocktail (Thermo Scientific) and phosphatase inhibitor cocktails 2 and 3 (Sigma-Aldrich). Whole cell lysates were resolved on a Criterion TGX 10% gel (Bio-Rad), transferred to an Immobilon PVDF membrane (Millipore, Billerica, MA), and probed with antibodies recognizing ERK1/2 and phosphorylated ERK1/2 (Cell Signaling Technologies, Danvers, MA). HRP-linked secondary antibodies against mouse and rabbit IgG (Cell Signaling Technologies) were used after probing with primary antibody. The blots were imaged using Clarity Western ECL Substrate (Bio-Rad) and Blue Devil autoradiography film (Genesse Scientific, San Diego, CA).

**TABLES**

**Table S1:** Oligonucleotide sequences used for *BRAF* c.1511_1517+2 dup study by PCR product length analysis

| **Oligonucleotide Name** | **Sequence (5' - 3')** | **PCR product size** |
| --- | --- | --- |
| c.1511_1517+2 dup _*Forward* | TCTGGAAAAGAGTAATTCACACA | 97pb |
| c.1511_1517+2 dup _*Reverse* | TGTTGAATGTGACAGCACCTACA |  |

**Table S2:** Result of PCR product length analysis designed to detect BRAF c.1511_1517+2 dup performed on LCH-derived gDNA from the french *BRAF*^V600E^-non mutated LCH children cohort.

| **Patient** | **BRAF c.1511_1517+2 dup** | **Gender** | **Age at diagnosis** | **Extension** | **Involved organs** | **First-line therapy** | **Response to first-line therapy** | **Recurrence** | **Biopsy conservation** | **% of tumor cells** |
| --- | --- | --- | --- | --- | --- | --- | --- | --- | --- | --- |
| **P10** | **neg** | M | 3.4 | RO- MS | Localized bone, skin, CNS | VLB-steroid | NAD | Yes | ffpe | 30 |
| **P11** | **neg** | M | 10.9 | RO- MS | Multifocal bone, pituitary | Wait and see | _ | Yes | ffpe | 30 |
| **P12** | **neg** | M | 10.8 | SS | Multifocal bone | Wait and see | _ | Yes | ffpe | 30 |
| **P13** | **neg** | F | 7.7 | lung+ | Multifocal bone, lung | VLB-steroid | ADB | No | ffpe | 15 |
| **P14** | **neg** | F | 2.6 | SS | Localized bone | Wait and see | _ | No | ffpe | 25 |
| **P15** | **neg** | M | 7.2 | SS | Localized bone | VLB-steroid | NAD | No | ffpe | 25 |
| **P16** | **neg** | M | 5.6 | SS | Localized bone | VLB-steroid | NAD | No | ffpe | 40 |
| **P17** | **neg** | M | 1.2 | RO- MS | Multifocal bone, skin | VLB-steroid | NAD | Yes | ffpe | 25 |
| **P18** | **neg** | F | 5.2 | SS | Localized bone | Wait and see | _ | No | ffpe | 25 |
| **P19** | **neg** | F | 11.5 | RO- MS | Multifocal bone, skin, pituitary | Wait and see | _ | No | ffpe | 40 |
| **P20** | **neg** | M | 3.3 | SS | Localized bone | Wait and see | _ | No | ffpe | 25 |
| **P21** | **neg** | M | 10.7 | SS | Multifocal bone | VLB-steroid | ADB | No | ffpe | 25 |
| **P22** | **neg** | M | 0.9 | SS | Localized bone | VLB-steroid | NAD | No | ffpe | 50 |
| **P23** | **neg** | M | 1.1 | SS | Localized bone | Wait and see | _ | No | ffpe | 25 |
| **P24** | **neg** | M | 0.1 | SS | skin | Wait and see | _ | No | ffpe | 50 |
| **P25** | **neg** | F | 5.0 | SS | Localized bone | Wait and see | _ | No | ffpe | 40 |
| **P26** | **neg** | F | 1.0 | SS | Multifocal bone | Wait and see | _ | No | ffpe | 25 |
| **P27** | **neg** | M | 3.5 | SS | Localized bone | Wait and see | _ | No | ffpe | 50 |
| **P28** | **neg** | F | 0.4 | SS | skin | Wait and see | _ | No | ffpe | 20 |
| **P29** | **neg** | F | 0.1 | SS | skin | Wait and see | _ | No | ffpe | 10 |
| **P30** | **neg** | F | 0.4 | RO- MS | Multifocal bone, skin | VLB-steroid | NAD | No | ffpe | 50 |
| **P31** | **neg** | F | 0.2 | RO- MS | Localized bone, skin, thymus, lymph nodes | Wait and see | _ | Yes | ffpe | 20 |
| **P32** | **neg** | M | 3.5 | SS | Multifocal bone | VLB-steroid | NAD | No | ffpe | 40 |
| **P33** | **neg** | F | 10.4 | SS | Multifocal bone | VLB-steroid | NAD | Yes | ffpe | 30 |
| **P34** | **neg** | M | 2.3 | SS | Multifocal bone | Wait and see | _ | No | ffpe | 30 |
| **P35** | **neg** | F | 0.3 | SS | lymph nodes | VLB-steroid | NAD | No | ffpe | 40 |
| **P36** | **neg** | F | 2.1 | RO- MS | Multifocal bone, skin | VLB-steroid | ADB | Yes | ffpe | 30 |
| **P37** | **neg** | F | 9.2 | SS | Localized bone | VLB-steroid | NAD | No | ffpe | 10 |

**Abbreviations:** ADB, active disease better; ffpe, formalin-fixed, paraffin-embedded F, female; M, male; MS RO-, multiple system LCH without risk organ involvement; NAD, non-active disease; SS, single system LCH; VLB, vinblastine

**FIGURES**

**
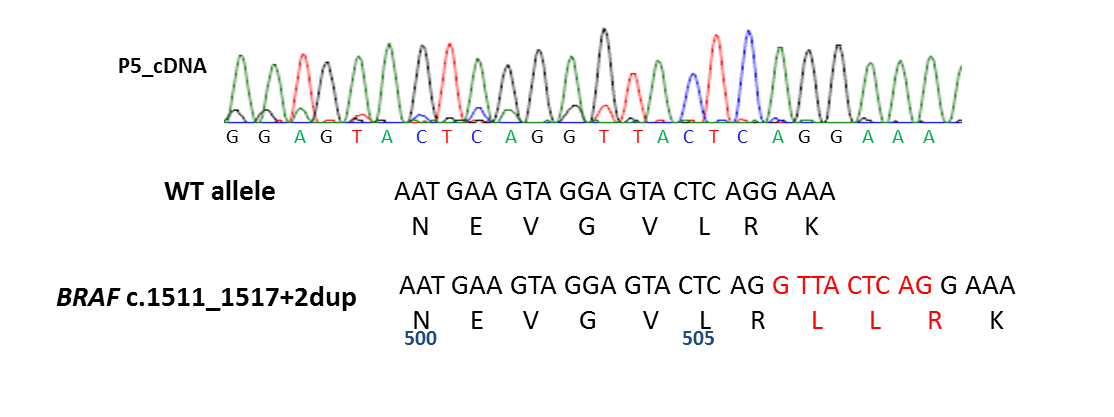
**

**Figure S1:** **cDNA sequencing of a LCH sample with the *BRAF* c.1511_1517+2dup mutation.** Sanger sequencing of P5 cDNA shows the inserted sequence which encodes to a mutant protein with insertion of 3 amino acids (p.Arg506_Lys507insLeuLeuArg)


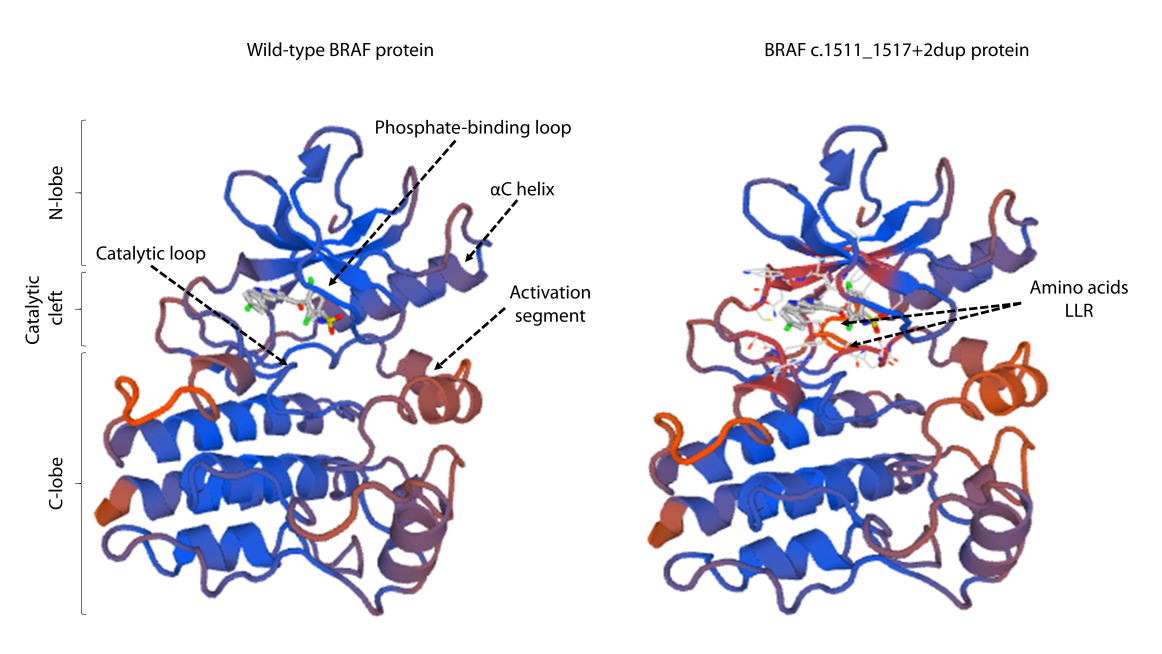


**Figure S2:** **Three-dimensional (3D) protein structure of BRAF c.1511_1517+2dup**. The 3D protein structure of the wild-type and mutated (*BRAF* c.1511_1517+2dup) human BRAF proteins were modeled and compared using SWISS-MODEL and Swiss-PdbViewer. Shown are ribbon diagrams of wild-type and mutated BRAF proteins depicting predicted structural changes resulting from the identified mutation, including alterations in the phosphate-binding loop and activation segment. Also highlighted are the LLR amino acids in the mutant protein. The 4rzv.2.A template was used to pattern and model both the wild-type and mutant 3D structures.

***
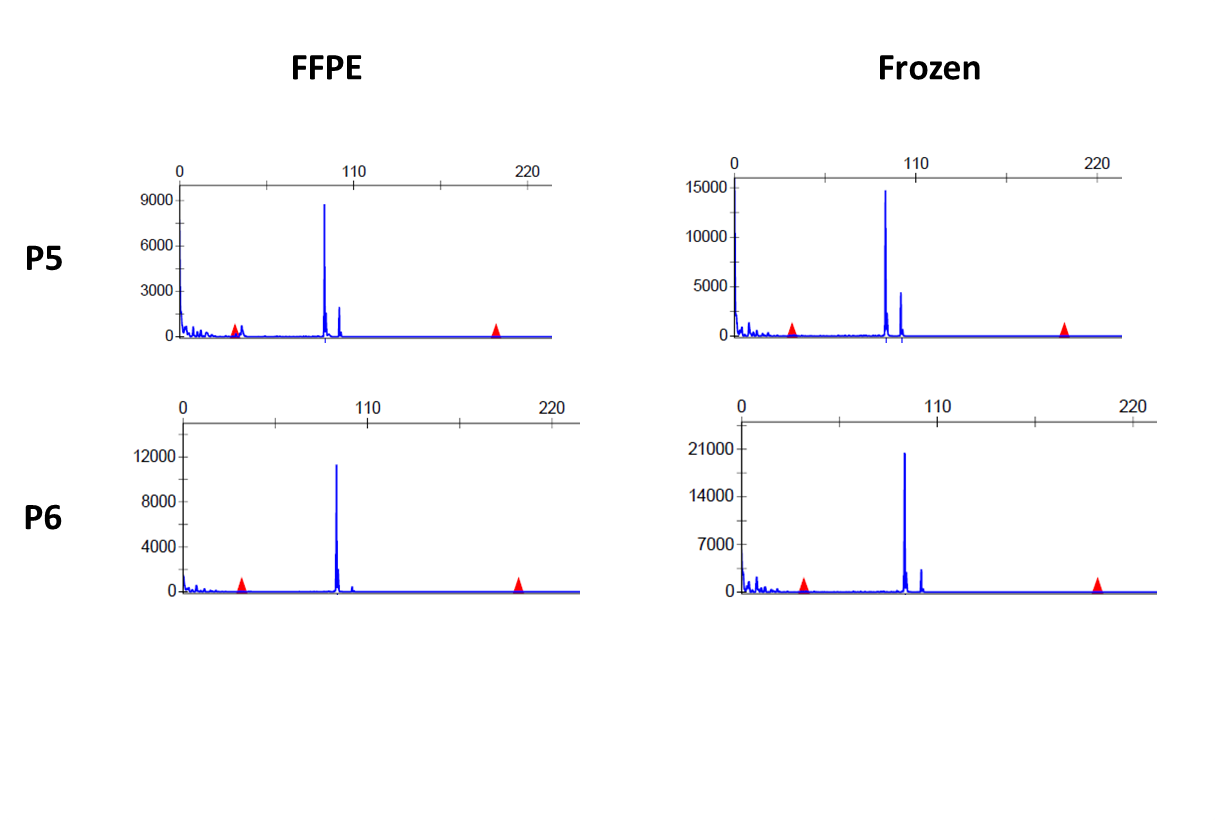
***

**Figure S3: P5 and P6 positive detection results of PCR product length analysis designed to detect BRAF c.1511_1517+2 duplication.** The appearance of a new peak corresponds to the mutated allele with a 9pb longer PCR product.

**SUPPLEMENTAL REFERENCES**

1. Colomba E, Hélias-Rodzewicz Z, Von Deimling A, Marin C, Terrones N, Pechaud D, et al. Detection of BRAF p.V600E mutations in melanomas: comparison of four methods argues for sequential use of immunohistochemistry and pyrosequencing. J. Mol. Diagn. 2013;15:94–100.

2. Brahimi-Adouane S, Bachet J-B, Tabone-Eglinger S, Subra F, Capron C, Blay J-Y, et al. Effects of endoplasmic reticulum stressors on maturation and signaling of hemizygous and heterozygous wild-type and mutant forms of KIT. Mol. Oncol. 2013;7:323–33.

3. Haroche J, Charlotte F, Arnaud L, von Deimling A, Hélias-Rodzewicz Z, Hervier B, et al. High prevalence of BRAF V600E mutations in Erdheim-Chester disease but not in other non-Langerhans cell histiocytoses. Blood. 2012;120:2700–3.

4. Berres M-L, Lim KPH, Peters T, Price J, Takizawa H, Salmon H, et al. BRAF-V600E expression in precursor versus differentiated dendritic cells defines clinically distinct LCH risk groups. J. Exp. Med. 2014;211:669–83.

5. Chakraborty R, Hampton OA, Shen X, Simko SJ, Shih A, Abhyankar H, et al. Mutually exclusive recurrent somatic mutations in MAP2K1 and BRAF support a central role for ERK activation in LCH pathogenesis. Blood. 2014;124:3007–15.

6. Chakraborty R, Burke TM, Hampton OA, Zinn DJ, Lim KPH, Abhyankar H, et al. Alternative genetic mechanisms of BRAF activation in Langerhans cell histiocytosis. Blood. 2016;128:2533–37.
